# A Potential Mechanism for Targeting Aggregates With Proteasomes and Disaggregases in Liquid Droplets

**DOI:** 10.3389/fnagi.2022.854380

**Published:** 2022-04-06

**Authors:** Emma Mee Hayes, Liina Sirvio, Yu Ye

**Affiliations:** ^1^Division of Neuroscience, Department of Brain Sciences, Imperial College London, London, United Kingdom; ^2^UK Dementia Research Institute at Imperial College London, London, United Kingdom

**Keywords:** liquid droplet, protein aggregation, ubiquitin proteasome pathway, disaggregase machinery, neurodegenaration, liquid-liquid phase separation, protein misfolding

## Abstract

Insoluble protein deposits are hallmarks of neurodegenerative disorders and common forms of dementia. The aberrant aggregation of misfolded proteins involves a complex cascade of events that occur over time, from the cellular to the clinical phase of neurodegeneration. Declining neuronal health through increased cell stress and loss of protein homeostasis (proteostasis) functions correlate with the accumulation of aggregates. On the cellular level, increasing evidence supports that misfolded proteins may undergo liquid-liquid phase separation (LLPS), which is emerging as an important process to drive protein aggregation. Studying the reverse process of aggregate disassembly and degradation has only recently gained momentum, following reports of enzymes with distinct aggregate-disassembly activities. In this review, we will discuss how the ubiquitin-proteasome system and disaggregation machineries such as VCP/p97 and HSP70 system may disassemble and/or degrade protein aggregates. In addition to their canonically associated functions, these enzymes appear to share a common feature: reversibly assembling into liquid droplets in an LLPS-driven manner. We review the role of LLPS in enhancing the disassembly of aggregates through locally increasing the concentration of these enzymes and their co-proteins together within droplet structures. We propose that such activity may be achieved through the concerted actions of disaggregase machineries, the ubiquitin-proteasome system and their co-proteins, all of which are condensed within transient aggregate-associated droplets (TAADs), ultimately resulting in aggregate clearance. We further speculate that sustained engagement of these enzymatic activities within TAADs will be detrimental to normal cellular functions, where these activities are required. The possibility of facilitating endogenous disaggregation and degradation activities within TAADs potentially represents a novel target for therapeutic intervention to restore protein homeostasis at the early stages of neurodegeneration.

## Introduction

Protein aggregates are observed in a range of neurodegenerative disorders, including Alzheimer’s, Parkinson’s, Huntington’s disease and amyotrophic lateral sclerosis ALS (ALS). Alzheimer’s disease is the most common form of dementia and is characterized by the presence of amyloid-β (Aβ) plaques (Knopman et al., 2021) and tau-containing neurofibrillary tangles (Goedert and Spillantini, 2017). Parkinson’s disease is a movement disorder, hallmarked by insoluble neuronal protein deposits called Lewy bodies that mainly consist of aggregated α-synuclein (αS) (Poewe et al., 2017). Huntington’s disease is typically characterized by chorea and psychiatric symptoms, in which expansion of repeat sequences (poly-glutamine or poly-Q) in the huntingtin protein (HTT) drives its aggregation (Tabrizi et al., 2020). The aggregation of several other proteins, such as TDP-43 and C9orf72, has been associated with ALS, a rare progressive neuromuscular disorder (Balendra and Isaacs, 2018). Interestingly, although these neurodegenerative disorders have distinct clinical presentations and are associated with specific proteins, the aggregation of misfolded proteins appears to be a common pathological mechanism (Soto and Pritzkow, 2018).

Compared with protein aggregation, the reverse process of aggregate disassembly by disaggregases and proteasomes has only recently gained attention. In this review, we will discuss the function of disaggregases and the ubiquitin (Ub)-proteasome system in restricting protein aggregation. We will examine how these components may assemble into liquid droplets, through a physical phenomenon called liquid phase condensation or liquid-liquid phase separation (LLPS), to potentially enhance their local activity. We propose that these enzymes and their co-proteins participate in the formation of putative “transient aggregate-associated droplets” (TAADs) which function as disaggregating/proteolytic centers to drive the disassembly of misfolded protein deposits. Enhancing the activity in such centers at the early stages of protein aggregation may represent a novel route to impede the progression of neurodegenerative disorders.

## Protein Aggregation

Misfolded proteins lack defined tertiary structures and tend to accumulate together, leading to protein aggregation. For example, both tau and αS are inherently disordered and lack defined tertiary structural features upon dissociation from their physiological binding partners (Vasili et al., 2019). Similarly, the expansion of repeat sequences, such as poly-Q in the *HTT* and poly-GA in the *C9orf72* gene, introduces unfolded protein sequences that form a critical feature in enabling their respective aggregation (Nonaka et al., 2018; Bonfanti et al., 2019). Other proteins, such as TDP-43 assemble into aggregates only upon specifically altered cellular conditions leading to protein misfolding, including LLPS-driven mechanisms (Watanabe et al., 2020).

**FIGURE 1:**
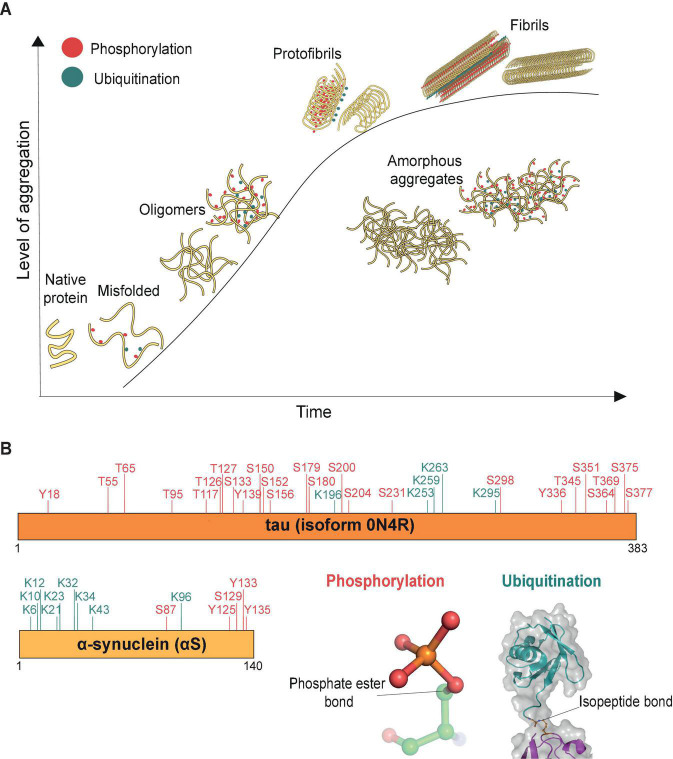
Aggregation process (time) of misfolded proteins. **(A)** Schematic representation of protein aggregation over time. The aggregation process (time) is represented by the *x*-axis, and the *y*-axis reflects the level of accumulated aggregates. Misfolded proteins deviate from their native conformations and start to assemble into oligomers (∼20–200 nm, Lobanova et al., 2021), mostly globular in shape. The initiation of the aggregation process may occur in conjunction with post-translational modifications, such as phosphorylation (red) and ubiquitination (cyan). As aggregation progresses, additional misfolded proteins accumulate and aggregates grow in size. Eventually structural rearrangements occur within the aggregates such that misfolded proteins start to organize into the ordered manner observed in protofibrils. Stable filamentous aggregates (fibrils) form from these highly ordered arrangements of misfolded proteins, now no longer soluble in their native environment. The various forms of aggregated proteins may sometimes assemble into amorphous aggregates, which have no distinct shapes and in which the individual misfolded proteins are not considered to have any ordered arrangements. **(B)** Both tau and α-synuclein (αS) are known to be post-translationally modified, shown here are the phosphorylation (red) and ubiquitination (cyan) sites identified by mass spectrometry in donor brain tissues of Alzheimer’s and Parkinson’s disease, respectively (Park et al., 2018; Zhang et al., 2019). Cartoon inserts detail molecular features of the covalent linkages associated with phosphorylation (pdb-id 4WZP) and ubiquitination (pdb-id 2JF5).

The aggregation process has been extensively studied for a range of misfolded proteins (Figure 1). Usually, misfolded protein monomers are thought to interact with each other and form globular oligomers that remain soluble (Breydo and Uversky, 2015). Oligomers can further aggregate and through structural rearrangements assemble into stable and highly organized filamentous aggregates (fibrils) or form amorphous aggregates that have no distinct higher-order structure (Figure 1A). Critically, these misfolded proteins and oligomers may act as templates or “seeds” for further aggregation to occur inside the cell (Goedert and Spillantini, 2017; Vasili et al., 2019). These aggregate seeds can also pass on to naïve cells, thus contributing to the spreading of pathology through the brain (Pecho-Vrieseling et al., 2014; Rauch et al., 2018, 2020). Studies in cellular and mouse models of pathology suggest that, compared with stable aggregates and fibrils, oligomers may be the more toxic aggregate species (Wegmann et al., 2016; De et al., 2019b), which easily penetrate lipid bilayers and cause cell stress (De et al., 2019a; Klenerman et al., 2021; Lobanova et al., 2021). The formation of aggregated fibrils, such as in neurofibrillary tangles and Lewy bodies, is reasoned likewise to be a potentially protective cellular mechanism to sequester the toxic oligomers (Kopeikina et al., 2012; Li and Haney, 2020).

In many cases, the aggregation of misfolded proteins is associated with post-translational modifications including phosphorylation and ubiquitination (Figure 1B). The role of phosphorylation, ubiquitination and many other post-translational modifications on tau and αS, such as sumoylation, nitration, O-GlcNacylation, acetylation and methylation, have been reviewed extensively for their roles in regulating the function of misfolded proteins through altering their physiochemical properties, turnover, structural conformation or interaction with their binding partners (reviewed in e.g., Zhang et al., 2019; Alquezar et al., 2020). Alterations in the phosphorylation status of misfolded proteins is believed to contribute to aggregate pathology. For example, hyperphosphorylation can cause tau to dissociate from microtubules, leading to microtubule destabilization and loss of neuronal cytoskeletal structure (Alonso et al., 2018). Additionally, tau phosphorylation at residues S202/T205 is considered a key pathological event of its aggregation (Simic et al., 2016). In Parkinson’s disease, αS is heavily phosphorylated at residue S129 (Fujiwara et al., 2002) and this modification is thought to accelerate aggregation of the protein (Karampetsou et al., 2017). Recent high-resolution cryo-EM studies have further found a high level of both ubiquitination and phosphorylation events on fibrils derived from post-mortem tauopathy and synucleinopathy donor samples (Arakhamia et al., 2020; Schweighauser et al., 2020), suggesting that both types of modifications are associated with aggregates.

A second aspect that may influence protein aggregation is LLPS, which describes a physical process whereby a macromolecular solution assumes a dense phase that resembles liquid droplets coexisting within a more dilute phase (Alberti et al., 2019). Demixing of intracellular macromolecules and assembly of liquid droplets may be influenced by the concentration and nature of the macromolecules and of the solution, all of which are factors that may determine whether phase separation occurs (principles of LLPS reviewed in Hyman et al., 2014). The phase state of droplets ranges from the “liquid-like” phase that readily fuse, coalesce and disassemble, to denser “gel-like” phases that are limited in exchange with the dilute phase but remain reversible. This phase can further progress into solid-like phases of hydrogels containing amyloid-like fibrils (review of phases in Peran and Mittag, 2020 and LLPS-relevant investigation techniques in Alberti et al., 2019). These hydrogels can be difficult to reverse without changes to the surrounding environment or interventions from other substrates. Droplets that do not readily exchange with the dilute phase or that are irreversible have been associated with a range of diseases (reviewed in Shin and Brangwynne, 2017; Alberti and Dormann, 2019). LLPS allows for the formation of membrane-less compartments (such as the nucleolus or centrosome) and enables cells to concentrate biomolecules at regions of interest, thus bringing relevant proteins in contact in a fluid and dynamic way (Hyman et al., 2014; Alberti and Hymannd, 2021). This process can also reduce variability and noise in protein concentration in cells (Klosin et al., 2020) and influence protein functions—for example, being in a more liquid phase would allow for increased molecular interactions while being in a more solid-like phase, although difficult to reverse, would sequester proteins from the cytosol (Hernandez-Vega et al., 2017; Olzmann and Carvalho, 2019). Emerging roles of LLPS in RNA processing and translation and neuroinflammation suggest that this physical process has important regulatory functions in biology (reviewed in Wolozin and Ivanov, 2019; Zbinden et al., 2020; Pakravan et al., 2021; Wang et al., 2021).

The formation of tau droplets is favored by hyperphosphorylation and pathological mutations such as P301S, P301L and A152T, which increase the likelihood of liquid-to-gel-like transition (Wegmann et al., 2018). While liquid droplets formed from tau proteins could sometimes be a physiological process to assist with microtubule polymerization in neurons (Hernandez-Vega et al., 2017), the increased density of tau also facilitates its aberrant aggregation (Ambadipudi et al., 2017). For HTT, it was shown that its aggregation-prone exon 1 fragment forms liquid droplets that became progressively resistant to 1,6-hexanediol, a compound that disrupts LLPS over time, indicative of the transition to a gel-like aggregate structure (Peskett et al., 2018). For αS-containing liquid droplets, a similar liquid-to-gel-like transition has been observed both *in vitro* and *in vivo*. The familial Parkinson’s disease mutations A53T and E46K in αS and S129-phosphorylated αS accelerate this transition, suggesting that LLPS may be a critical step in aggregation (Ray et al., 2020). The important next step is to unveil the molecular features of LLPS-driven aggregation of misfolded proteins in a physiological context.

## Reversing Protein Aggregation

**FIGURE 2:**
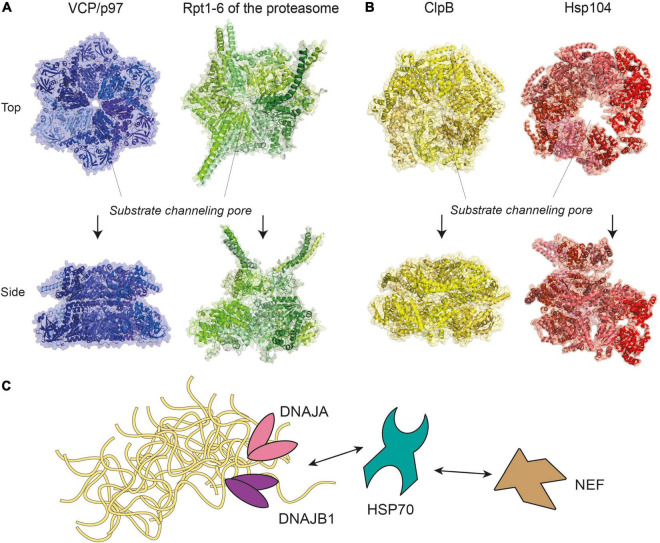
Molecular representations of enzyme complexes with protein unfolding/refolding activity. **(A)** Metazoan VCP/p97 (pdb-id 5C1A) (Hanzelmann and Schindelin, 2016), a homohexamer of the same superfamily and colored in different shades of blue, shown next to the six ATPases of the 19S proteasome subparticle (see Figure 3), a heterohexamer consisting of Rpt1-6 (pdb-id 5GJR) (Huang et al., 2016). **(B)** Prokaryotic ClpB (pdb-id 6QS4) (Deville et al., 2019) and its yeast ortholog Hsp104 (pdb-id 5KNE) (Yokom et al., 2016) are homohexamers of the AAA+ ATPase superfamily. For clarity, identical subunits within the same complex are colored with different shades of the same color scheme. The positions of substrate channeling pores are indicated with dotted lines. **(C)** A model of the HSP70 disaggregase machinery, where HSP70 work together with DNAJA, DNAJB1, and nucleotide exchange factor (NEF) to target aggregates of various sizes. Double-headed arrows represent reversible interactions between proteins.

Disaggregases, such as the metazoan VCP (also known as p97), and the 70 kDa heat shock proteins (HSP70) disaggregase machinery, are a group of enzymes that utilize ATP to reverse protein aggregation, refolding aggregates back into monomers in their native conformations (Desantis and Shorter, 2012). These enzymes may target aggregates alone or associate with additional co-proteins that assist with enzymatic functions. VCP/p97 belongs to the AAA+ ATPase superfamily (Puchades et al., 2020), which form characteristic hexameric ring complexes that couple ATP hydrolysis with mechanical work (Figure 2A). Other members of this superfamily include subunits Rpt1-6 of the 19S proteasome regulatory particle, prokaryotic ClpB and its yeast ortholog Hsp104 (Figure 2B), whose disaggregation activity was reported nearly three decades ago (Parsell et al., 1994). A recent report further reveals a metazoan AAA+ ATPase disaggregase, Skd3, a mitochondrial protein that is able to solubilize αS aggregates and is distantly related to ClpB (Cupo and Shorter, 2020). The ability to restrict and reverse protein aggregation appears to be an activity that has been conserved in evolution.

### VCP/p97 and Disaggregation

VCP/p97 was found to associate with the HSP70 disaggregase machinery (discussed below) and engage with tau and HTT aggregates in post-mortem brain tissues (Hirabayashi et al., 2001; Darwich et al., 2020). VCP/p97 plays a pivotal role in endoplasmic reticulum (ER)-associated degradation by pulling misfolded proteins from ER membranes for proteasomal degradation (Wolf and Stolz, 2012). Apart from assisting with degradation of misfolded proteins, VCP/p97 also contributes to other cellular processes including chromatin remodeling, transcriptional regulation and the DNA damage response (Vaz et al., 2013).

Previous studies found a very high level of expression of VCP/p97 in mammalian cells, underscoring its importance in proteostasis functions (Peters et al., 1990; Zeiler et al., 2012). Compared with other members of the AAA+ ATPase superfamily, VCP/p97 lacks motifs within the catalytic domain that facilitate binding and guidance of substrates through the central pore (Hanzelmann and Schindelin, 2017; Saffert et al., 2017). Instead, VCP/p97 associates with co-factors Ufd1 and Npl4, which aid in the recognition, binding and transfer of ubiquitinated substrates to VCP/p97 (Blythe et al., 2017; Bodnar and Rapoport, 2017). Mutations on VCP have been linked to several forms of neurodegenerative disorders (Watts et al., 2004; Johnson et al., 2010; Meyer and Weihl, 2014; reviewed in Sun and Qiu, 2020) and endogenous VCP associates with protein inclusions in both Huntington’s disease and Lewy body dementia (Hirabayashi et al., 2001).

The VCP/Ufd1/Npl4 machinery targets ubiquitinated substrates and is linked with proteasomal responses to aggregated proteins. For instance, knocking out components of the VCP/p97 machinery shows impeded degradation of ubiquitinated HTT (Higgins et al., 2020). Similarly, proteasome-mediated degradation of TDP-43 aggregates is impeded by the VCP inhibitor NSM-873 (van Well et al., 2019). A recent study further demonstrates that wild-type VCP/p97 is capable of disassembling tau aggregates derived from Alzheimer’s disease donors, but a frontotemporal dementia-linked pathological D395G mutation in VCP showed markedly reduced disaggregation activity (Darwich et al., 2020). This provides evidence for the direct involvement of VCP/p97 activity in a clinically observed neurodegenerative disorder. A strong association exists between the VCP machinery and the proteasome system to enable unfolding-degradation of ubiquitinated aggregates, presumably in a sequential manner. The highly specialized roles of VCP, Ufd1, Nlp4 and the proteasome may provide better control and specificity for each of the successive steps in targeting the aggregates for removal.

### 70 kDa Heat Shock Proteins Family and Disaggregases

The 13-member HSP70-family of molecular chaperones participates in the folding of newly synthesized proteins, assists with the translocation of polypeptides into organelles, and the disassembly of stable complex structures. Many of the cellular homeostatic protein folding functions are thought to be carried out by the constitutively expressed HSP70-family member HSC70, while expression of the HSP70 protein is induced by stress and accumulation of misfolded proteins (Rosenzweig et al., 2019). HSP70 recognizes misfolded proteins by binding to hydrophobic protein stretches such as the tetra-repeat domain of tau (Kundel et al., 2018) or the N-terminal region of αS (Tao et al., 2021), inhibiting their aggregation propensity (Klucken et al., 2004; Dedmon et al., 2005; Kundel et al., 2018; Tao et al., 2021). The binding of HSP70 to Aβ and HTT oligomers is also shown to prevent further aggregation, though HSP70 alone is unable to disassemble Aβ fibrils (Muchowski et al., 2000; Evans et al., 2006).

Chaperones of the HSP70-family form a disaggregase system in metazoans by recruiting additional co-chaperones called J-proteins and a nucleotide exchange factor (NEF), such as Apg2 and HSP110 (Rosenzweig et al., 2019). This HSP70/J-protein/NEF combination is hereafter referred to as the HSP70 disaggregation machinery (Figure 2C). By engaging different classes of J-proteins, the HSP70 chaperone forms transient complexes that disassemble aggregates of a range of sizes with assistance from the NEF (Nillegoda et al., 2015). Similarly, the HSC70 chaperone could disassemble αS fibrils *in vitro* by associating with a J-protein (DNAJB1) and a NEF (Apg2), decreasing aggregate toxicity by ∼10% (Gao et al., 2015). However, more recent data shows that disaggregation of brain-derived tau filaments via the HSC70/DNAJB1/Apg2 machinery produced oligomeric tau that was more seeding-competent in HEK293 cells (Nachman et al., 2020). These results indicate that whether toxicity increases or decreases following the disassembly activity may depend on how completely the disaggregation occurs.

### C-Terminus of Hsc70-Interacting Protein and Aggregate Degradation

**FIGURE 3:**
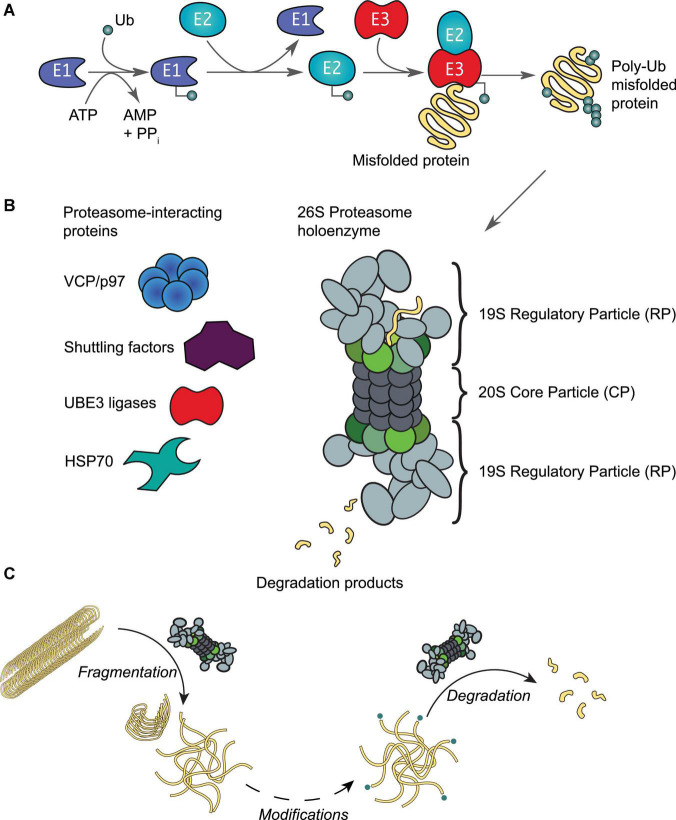
The ubiquitin-proteasome system. **(A)** Protein ubiquitination is initiated by an E1 activating enzyme, which charges Ub to its active site cysteine residue in an ATP-dependent manner. This activated Ub is then transferred to the active site cysteine of an E2 conjugating enzyme, which with support from an E3 ligating enzyme, results in protein ubiquitination. Ubiquitinated proteins are recognized by proteasomes, leading to the unfolding and degradation of the substrate. **(B)** Schematic representation of the 26S proteasome holoenzyme. This enzyme complex consists of a 20S core particle (CP), capped by 19S regulatory particles (RP). Both the CP and RP may exist as free particles or assembled into a holoenzyme. Rpt1-6 of the RP are colored in shades of green as in Figure 2A. A number of proteins interact reversibly to assist with various proteasomal functions as required by the host cell. **(C)** In addition to protein degradation, the proteasome holoenzyme is also able to fragment stable fibrils into smaller fragments (< 500 nm) such as oligomers (Cliffe et al., 2019); as well as capable of degrading oligomers that are ubiquitinated at their N-termini by e.g., UBE2W (Ye et al., 2020).

The HSP70 system interacts with C-terminus of Hsc70-interacting protein (CHIP), a Ub E3 ligase (see Figure 3A), and targets aggregates for proteasomal degradation. CHIP facilitates substrate ubiquitination through its C-terminal catalytic domain, while its N-terminal domain enables interactions with HSP70 (Zhang et al., 2020). Interactions with the disaggregation machinery appears critical for CHIP activity, as aggregate-bound chaperones are required for efficient ubiquitination (Qian et al., 2006; Tetzlaff et al., 2008; Kalia et al., 2011). Our recent *in vitro* study further suggests that the binding of misfolded proteins such as tau may directly enhance the activity of CHIP (Zuo et al., 2020). Whether misfolded proteins and aggregates are refolded or degraded is thought to be dependent on the ratio of the disaggregation machinery to CHIP. HSP70 and other heat shock proteins are expressed at much higher levels than CHIP (Meacham et al., 2001), suggesting a potential cellular preference for refolding over degradation. However, upon elevated CHIP expression or prolonged substrate-HSP70 interactions, a larger fraction of substrates become ubiquitinated by CHIP, tagging them for degradation (Qian et al., 2006).

Many misfolded proteins and their aggregates are substrates of CHIP. It was shown that CHIP ubiquitinates HSP70-bound αS and accelerates the degradation of αS aggregates, leading to reduced aggregate toxicity (Shin et al., 2005; Tetzlaff et al., 2008). CHIP-mediated tau ubiquitination and degradation could be augmented in HeLa cells and in a mouse model of tauopathy, upon induced endogenous HSP70 expression through inhibition of HSP90 (Petrucelli et al., 2004; Dickey et al., 2007). Toxicity induced by Aβ and HTT is reduced by CHIP ubiquitination and subsequent proteasomal degradation in mammalian cells and mouse disease models (Jana et al., 2005; Miller et al., 2005; Kumar et al., 2007). In a rodent Huntington’s disease model, the loss of CHIP accelerates pathological phenotypes such as HTT aggregation and neurodegeneration (Miller et al., 2005). It therefore appears that increasing CHIP activity would be beneficial for restricting protein aggregation in cells.

## The UB-Proteasome System in Reversing Protein Aggregation

### The Proteasome System

Aberrant loss of proteasomal degradation activity is observed in Alzheimer’s, Parkinson’s and Huntington’s disease (Keller et al., 2000; Furukawa et al., 2002; McNaught et al., 2002; Seo et al., 2004; Diaz-Hernandez et al., 2006; Thibaudeau et al., 2018). Substrates destined for degradation are normally conjugated with multiple Ub moieties through a sophisticated enzymatic cascade involving E1, E2, and E3 enzymes (Figure 3A; Komander and Rape, 2012). The concerted actions of these enzymes ensure that virtually all biological processes involve protein ubiquitination events at some point (Swatek and Komander, 2016; van Wijk et al., 2019). The diversity in cellular outcomes is believed to be dictated by distinct forms of ubiquitination of the substrate (reviewed by e.g., Komander and Rape, 2012; Swatek and Komander, 2016; Oh et al., 2018; van Wijk et al., 2019). Both tau and αS have been observed with typical Ub modifications that include mono-, multiple- and polyubiquitination (Zhang et al., 2019; Alquezar et al., 2020). Enzymes of the UPS, along with Ub moieties and chaperones, are abundantly detected in insoluble aggregate structures, such as Lewy bodies and neurofibrillary tangles (Garcia-Sierra et al., 2012; Zhang et al., 2019; Arakhamia et al., 2020; Schweighauser et al., 2020), suggesting an important role for the Ub-proteasome system in protein aggregation. In fact, several other E3 enzymes of the Ub system have been implicated in neurodegeneration (reviewed in Zheng et al., 2016; Upadhyay et al., 2017). Canonically, ubiquitinated proteins are recognized by the 26S proteasome holoenzyme (Collins and Goldberg, 2017; Marshall and Vierstra, 2019). This holoenzyme consists of a barrel-shaped 20S core particle capped on one or both ends by 19S regulatory particles (Figure 3B; Budenholzer et al., 2017; Bard et al., 2018). Unfolded proteins are processed by subunits with proteolytic activities that reside in the 20S core particle (Rennella et al., 2020; Coleman et al., 2021). The regulatory particle is responsible for substrate recognition, deubiquitination, and ATP-dependent substrate unfolding that enables subsequent translocation through the channel pore (Lu et al., 2017). The base of the regulatory particle, including the hexameric ATPase, is suggested to have some chaperone activity (Braun et al., 1999).

Early works found that aggregates impaired protein degradation via the UPS in HEK293 cells (Bence et al., 2001; Bennett et al., 2005). More recent studies have shown that oligomers in particular have a strong inhibitory effect on the activity of the proteasome by e.g., preventing substrate translocation and protein degradation (Kristiansen et al., 2007; Myeku et al., 2016; Thibaudeau et al., 2018). In agreement, engineered proteasomes with a constitutively open channel pore that facilitates access to the core particle increases tau aggregate degradation and cell survival to toxic insults (Choi et al., 2016). We have recently demonstrated that the proteasome holoenzyme possesses a novel fibril-fragmenting activity, capable of breaking stable fibrils into smaller, more toxic fragments (Cliffe et al., 2019). In a follow-up study, we further show that a single Ub modification at the N-terminus of tau or αS, similar to ubiquitination events catalyzed by the E2 enzyme UBE2W, enables their oligomers to be potently degraded by the proteasome holoenzyme (Ye et al., 2020), while unmodified oligomers are resistant to degradation (Iljina et al., 2016). It may therefore be plausible that proteasomes first break fibrils into smaller entities and that additional enzymes are recruited to assist the removal of oligomers in a Ub-dependent manner (Figure 3C). Similar to observations in disaggregases, it is plausible that proteasome activity may aggravate or decrease toxicity depending on how completely aggregate degradation occurs.

### Proteasome-Interacting Proteins in Mediating Aggregate Degradation

Several studies have indicated that the proteasome interacts with components of HSP70 and VCP disaggregase machineries (Guerrero et al., 2008; Besche et al., 2009). A study of proteasome-associated proteins isolated from the human brain suggests that proteasomes bind to both HSP70 and VCP (Tai et al., 2010). Possibly, the interaction between proteasomes and disaggregases facilitates degradation of terminally misfolded proteins and aggregates through the sequential action of disaggregation-degradation (Figure 3C). Similarly, co-proteins known to interact with the proteasome, such as UBQLN2 and UBE3C (discussed below), may also assist aggregate removal in a concerted manner.

**UBQLN2** is a proteasome shuttling factor, which contains an N-terminal domain that interacts with the proteasome, and a C-terminal domain to which ubiquitinated and misfolded proteins may bind (Finley, 2009). The interaction between proteasome and UBQLN2 appears critical for neurons, as deletion of its N-terminal domain increases UBQLN2 punctate formation and neuronal toxicity (Sharkey et al., 2018). Mutations in UBQLN2 are implicated in familial ALS and have been found to decrease proteasome-mediated aggregate degradation (Deng et al., 2011; Chang and Monteiro, 2015; Hjerpe et al., 2016). Interestingly, UBQLN2 appears to preferentially associate with larger protein aggregates over monomers (Zhang et al., 2021). This seems consistent with the observation that deletion of *dsk2*, the yeast homolog of *UBQLN2*, has no effect on early HTT aggregation but was required once intracellular protein deposits of aggregated proteins known as inclusion bodies have formed (Chuang et al., 2016).

Interactions between protein aggregates and HSP70 are facilitated by UBQLN2. Previous studies show that UBQLN2 is activated by HTT or poly-GA aggregates binding to HSP70 (Hjerpe et al., 2016; Zhang et al., 2021). This interaction is abolished in the presence of ALS/frontotemporal dementia-linked mutations in UBQLN2 or knock-down of CHIP (Zhang et al., 2021). Plausibly, aggregates are targeted by CHIP-HSP70-UBQLN2 in an ordered manner to ensure their efficient degradation at the proteasome. As HSP70 can coat protein aggregates (Whiten et al., 2018; Nachman et al., 2020), recruiting UBQLN2 could provide a mechanism for HSP70 to deal with larger ubiquitinated and disassembly-resistant aggregates.

**UBE3C** is a Ub E3 ligase that is recruited to the proteasome when protein misfolding is induced (Gottlieb et al., 2019). Hul5, its yeast ortholog, targets misfolded proteins following heat shock ubiquitination response and removes low-solubility proteins and aggregates during stress (Fang et al., 2011). Overexpressing Hul5 partially rescues the accumulation of protein aggregates when the protein quality control network is compromised (Eisele et al., 2021), suggesting an important role for Hul5’s ubiquitination activity at the proteasome in impeding protein aggregation. The protein quality control function of Hul5 is recapitulated in human UBE3C, which is recruited to aggresomes via HSP70 interaction and promotes degradation of its substrates (Mishra et al., 2009). A number of mutations on UBE3C that either render the ligase inactive or lead to reduced enzymatic activity are linked to autism spectrum disorder (Ambrozkiewicz et al., 2020; Singh and Sivaraman, 2020). The activity of UBE3C assists proteasomal degradation by enhancing the complete removal of partially degraded proteins (Chu et al., 2013) and prevents ubiquitinated substrates from binding stalled proteasomes (Besche et al., 2014).

Perhaps with some redundancy, UBE3A and UBE3B are also members of the UBE3 ligase family and interact reversibly with the proteasome to assist its function. UBE3A mutations affecting proteasomal binding or activity have been linked to Angelman syndrome (Yi et al., 2017; Kuhnle et al., 2018). In neurons, UBE3A is found at axon terminals and dendritic spines (Burette et al., 2018), in agreement with its role in assisting proteasome functions in regulating neural processes (Hsu et al., 2015; Kim et al., 2016). Similarly, UBE3B is found to regulate the branching of dendrites and metabolic processes, and knock-down of UBE3B renders cells sensitive to oxidative stress (Svilar et al., 2012). It is plausible that evolution has created partial redundancy and partial specificity in UBE3 ligases to enable distinct outcomes for ubiquitination at the proteasome during stress.

### Cross-Talk Between Proteasomal and Autophagosomal Degradation

A second route for degradation of eukaryotic proteins is through the autophagy-lysosome pathway, which shares links to the UPS (Dikic, 2017; Varshavsky, 2017). The flux of this pathway is stimulated upon induction of specific cellular stress conditions such as starvation or protein aggregation (Chen et al., 2019). Substrates targeted by the autophagy-lysosome pathway are enclosed in vesicles known as autophagosomes and are degraded following their fusion with lysosomes (Runwal et al., 2019). Selective degradation of protein aggregates via autophagy, known as aggrephagy, plays a critical role in restricting their accumulation in cells (Lamark and Johansen, 2012). Like proteasome activity, there is evidence of autophagic dysfunction in neurodegenerative disorders (Menzies et al., 2017). There is significant cross-talk between these two systems (reviewed by e.g., Korolchuk et al., 2010; Bustamante et al., 2018)—for example in SH-SY5Y neuroblastoma cells, proteasome inhibition via MG132 activates the transcription factor EB, a master regulator of autophagy that increases the expression of genes involved in autophagy activation and increases autophagosome biogenesis (Li et al., 2019). In HEK293 cells, loss of HSP70 or its cofactor HSP110 is found to induce autophagy and reduce proteasome activity (Feleciano et al., 2019).

The scaffold protein p62 is known to mediate cross-talk between the UPS and the autophagy-lysosome pathway (Bustamante et al., 2018). p62 serves as a signaling hub for diverse cellular processes and functions as an autophagy receptor to enable degradation of ubiquitinated proteins (Katsuragi et al., 2015). The UBA domain of p62 interacts with LC3, a key protein involved in autophagosome formation and thereby bridging between substrate ubiquitination and autophagosomal degradation (Pankiv et al., 2007). An early study in HeLa cells demonstrates p62 co-localizes with ubiquitinated protein aggregates and along with LC3 forms a shell surrounding HTT protein aggregates (Bjorkoy et al., 2005). Expectedly, loss of p62 increases HTT aggregation-mediated cell death. More recently, it is found that the loss of two Ub receptors of the proteasome led to the accumulation of ubiquitinated proteins and activates autophagy via p62/LC3 mechanisms (Demishtein et al., 2017). This suggests existence of a balance between proteasomal and autophagosomal degradation activities, which is mediated by p62.

## The Role of Liquid-Liquid Phase Separation in Protein Aggregation

### The Proteasome System and Disaggregases Are Involved in Liquid Droplets

Proteasomes are highly abundant in mammalian cells and are distributed throughout the cytosol and the nucleus (e.g., Brooks et al., 2000; Pack et al., 2014; Zhang et al., 2018). Previous studies have shown that starvation and nutrient depletion in yeast and plant cells induce proteasomes to assemble into storage granules (Gu et al., 2017; Marshall and Vierstra, 2018). In mammalian cells, osmotic shock induces the accumulation of proteasomes into foci structures (Lafarga et al., 2002). Our data show that such cellular bodies are assembled promptly upon stress and do not appear to be dependent on cytoskeletal transport (Zhang et al., 2018). Recently, Yasuda et al. (2020) demonstrated that these stress-induced proteasome foci are assembled through LLPS and contain denser degradation activity than the surrounding cellular environment. These foci also stain positive for VCP/p97, UBE3A and Ub-modified substrates, and are together suggested to form individual proteolytic centers in the cell (Yasuda et al., 2020). Inhibition of VCP/p97 increases foci size, whereas inhibition of ubiquitination activity prevents foci formation, and knock-out of UBE3A reduces foci number. VCP/p97 and proteasomes have also been reported to co-localize in stress granules induced by oxidative stress (Turakhiya et al., 2018).

Molecular chaperones and components of the disaggregase machinery also appear to assemble into or associate with cellular bodies that act under LLPS principles. UBQLN2 co-localizes with stress granules and undergoes LLPS under *in vitro* conditions as well as in HeLa cells, where its HSP70 interaction domain is critical for liquid droplet formation (Hjerpe et al., 2016). The VCP machinery contributes to clearing stress granules, with mutations on VCP/p97 delaying the disappearance of stress granules and stress recovery (Buchan et al., 2013). In yeast cells, Hsp70 and its co-chaperone Hsp104 co-localize with stress granules and are essential for post-stress recovery (Cherkasov et al., 2013). In HeLa cells, accumulated TDP-43 was found to be held in cytoprotective nuclear bodies by HSP70 (Gu et al., 2021). Similarly, HSP70 associates with and maintains ribonuclear granules in a liquid-like state (Ganassi et al., 2016; Mateju et al., 2017). In an elegant study by Frottin et al. (2019), misfolded proteins were found to enter LLPS-dependent granules to prevent irreversible aggregation. HSP70 was required to recover proteins from the stress-induced granules to allow their refolding or degradation.

A recent study showed that proteasome particles, Ub E1, E2, E3 enzymes and deubiquitinases are found in liquid droplets in the nucleus, as was p62, the essential component of these droplets (Fu et al., 2021). These LLPS features were dependent on the interaction between p62 and polyubiquitin chains as well as p62 self-interaction (Sun et al., 2018; Zaffagnini et al., 2018). The binding of ubiquitinated substrates appears to heavily influence the ability of p62 to promote protein phase separation (Zaffagnini et al., 2018). Upon proteolytic stress in HeLa cells, HSP70, CHIP and HSP90, another heat shock protein, is recruited to the p62-essential nuclear foci enriched in proteasome particles to enhance the degradation of ubiquitinated substrates (Fu et al., 2021). These foci are suggested to be catalytic centers for degradation of misfolded proteins resulting from heat and oxidative stress. Together, the multiple protein interactions involving ubiquitinated substrates, HSP70, proteasomes and their co-proteins may provide a rationale for how liquid droplets coordinate to maintain proteostasis in response to aggregation of misfolded proteins.

### Could Liquid-Liquid Phase Separation Be a Novel Mechanism to Concentrate Disaggregation and Degradation Activities?

**FIGURE 4:**
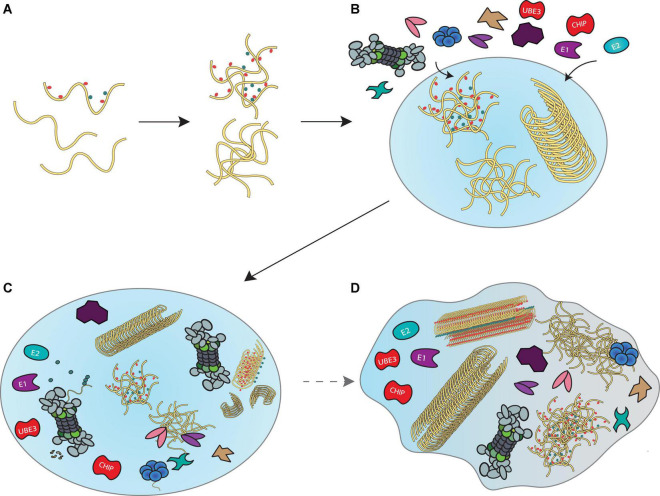
A model for disaggregation and degradation reactions mediated by liquid droplets. **(A)** Proteins that misfold inside a cell begin to accumulate and form liquid droplets through LLPS principles. **(B)** Recruitment of the ubiquitin-proteasome system, VCP and HSP70 disaggregation machineries and co-proteins that support their functions enable locally concentrated enzymatic activities in liquid droplets to prevent further accumulation of misfolded proteins. **(C)** Formation of such “transient aggregate-associated droplets” (TAADs) enable concerted actions of fibril disassembly, ubiquitination and unfolding of aggregates and substrate degradation to occur within a dedicated environment. **(D)** Prolonged stalling of the various enzymes in the TAADs through disassembly- and degradation-resistant aggregation mechanisms may be associated with gradual phase shift towards a more gel-like state that could be relevant to pathology.

Given that components of the disaggregase machineries, proteasomes and their co-proteins have all been found in liquid droplets, it is tempting to speculate that a “transient aggregate-associated droplet” (TAAD) is assembled in response to accumulating misfolded proteins (Figure 4). These TAADs may be initiated through the presence of misfolded protein condensates, to which chaperones, proteasomes or their co-proteins would nucleate. Additional components and interacting partners of disaggregase and proteasome systems would also be recruited. The existence of such droplets would facilitate the concerted actions of ubiquitination, disaggregation, refolding/unfolding and degradation to take place locally, ensuring a high density of disassembly- and proteolytic activity to occur without compromising homeostatic cellular processes outside the TAAD. An elegant cryo-electron tomography study in nematodes reports that poly-GA aggregates recruit proteasomes to form foci structures with aggregates (Guo et al., 2018). Since these aggregates do not affect 26S proteasome expression, it follows that proteasomes are depleted from other cellular regions—indeed proteasome concentration is ∼ 30 fold higher within poly-GA aggregates than the cellular surroundings. Others and we have further reported the super-resolution imaging of proteasome foci in live cells (Gu et al., 2017; Zhang et al., 2018), describing a high local density of these molecular degradation machines under stress. Similar recruitment of proteasomes to aggresomes (Hao et al., 2013) and granules (Frottin et al., 2019) has been observed, further underscored by our *in vitro* experiments that demonstrated direct proteasomal actions on both large fibrils and small Ub-modified oligomers (Cliffe et al., 2019; Ye et al., 2020).

The TAADs may only assemble briefly as a result of stress-related emergencies, since the prolonged engagement of disaggregases and proteasomes would neglect their canonical roles in proteostasis. As mentioned in the second section, aggresomes and other large, end-stage aggregates and fibrils may be neuroprotective and less toxic than oligomers. However, it is possible that fibrils could indirectly affect disaggregases and the proteasome system by depleting their enzymatic activity from elsewhere in the cell, so that they cannot fulfill their normal function in proteostasis. It may further be possible that a phase change to a more gel-like state could trap these enzymes in the TAADs, which would further aggravate protein aggregation. Current methods using fluorescence recovery after photobleaching (FRAP) to determine how rapidly proteins found within the droplet exchange with those outside, combined with determining droplet fluidity by measuring how stable they are in the presence of 1,6-hexanediol treatment, will enable examination of the phase of TAADs.

## Concluding Remarks

We have discussed the ability of the disaggregase and the proteasome systems to assemble into liquid droplets, proposing the concept of TAADs to allow these systems to act in a concerted manner to remove aggregates. These putative droplets may provide an “emergency solution” to cell stress, temporarily redistributing enzymes needed for proteostasis in the cell. Aggregation-prone proteins and disaggregases appear to act in an LLPS-driven manner, enabling cells to enhance the disassembly of aggregates within droplet structures. A critical next step is to quantitatively examine the early stages of neurodegeneration for changes in droplet size, fluidity, composition and the local disaggregase/degradation activity.

Recent technical developments such as “optoDroplet,” takes advantage of optogenetics to precisely control and study phase transitions within cells (Shin et al., 2017). The construct in optoDroplet consists of an intrinsically disordered protein region fused to a light-sensitive protein that self-interacts upon light exposure, thus enabling light intensity to tune droplet formation in cells. Potentially, a protein of interest acting under LLPS principles may be fused to the optoDroplet construct and brought into droplet formation in a photo-controllable manner. This technique adequately complements the canonical methods for assessing LLPS in cells such as 1,6-hexanediol (McSwiggen et al., 2019), FRAP (Alberti et al., 2019) or alteration of buffer conditions (Wei et al., 2017; Schuster et al., 2018). Furthermore, combining optoDroplet with CRISPR technology (Nihongaki et al., 2017) would allow researchers to perform genome-wide screens to further identify proteins that act under LLPS principles. Currently, it is difficult to determine whether disaggregase and proteasome droplets are beneficial for aggregate removal or detrimental to overall cell proteostasis. However, by utilizing both established and novel techniques for investigating LLPS and its contribution to protein aggregation, therapeutic interventions may be developed to restore protein homeostasis at the early stages of neurodegeneration.

## Author Contributions

EMH and LS performed the literature review. YY conceptualized the manuscript and supervised the project. EMH and YY wrote the original draft. All authors contributed manuscript writing and editing.

## Conflict of Interest

The authors declare that the research was conducted in the absence of any commercial or financial relationships that could be construed as a potential conflict of interest.

## Publisher’s Note

All claims expressed in this article are solely those of the authors and do not necessarily represent those of their affiliated organizations, or those of the publisher, the editors and the reviewers. Any product that may be evaluated in this article, or claim that may be made by its manufacturer, is not guaranteed or endorsed by the publisher.

## References

[B1] Alberti S. Dormann D. (2019). Liquid-liquid phase separation in disease. *Annu. Rev. Genet.* 53 171–194. 31430179 10.1146/annurev-genet-112618-043527

[B2] Alberti S. Gladfelter A. Mittag T. (2019). Considerations and challenges in studying liquid-liquid phase separation and biomolecular condensates. *Cell* 176 419–434. 10.1016/j.cell.2018.12.035 30682370 PMC6445271

[B3] Alberti S. Hymannd A. A. (2021). Biomolecular condensates at the nexus of cellular stress, protein aggregation disease and ageing. *Nat. Rev. Mol. Cell Biol.* 22, 196–213. 10.1038/s41580-020-00326-6 33510441

[B4] Alonso A. D. Cohen L. S. Corbo C. Morozova V. ElIdrissi A. Phillips G. (2018). Hyperphosphorylation of Tau associates with changes in its function beyond microtubule stability. *Front. Cell. Neurosci.* 12:338. 10.3389/fncel.2018.00338 30356756 PMC6189415

[B5] Alquezar C. Arya S. Kao A. W. (2020). Tau post-translational modifications: dynamic transformers of tau function, degradation, and aggregation. *Front. Neurol.* 11:595532. 10.3389/fneur.2020.595532 33488497 PMC7817643

[B6] Ambadipudi S. Biernat J. Riedel D. Mandelkow E. Zweckstetter M. (2017). Liquid-liquid phase separation of the microtubule-binding repeats of the Alzheimer-related protein Tau. *Nat. Commun.* 8:275. 10.1038/s41467-017-00480-0 28819146 PMC5561136

[B7] Ambrozkiewicz M. C. Cuthill K. J. Harnett D. Kawabe H. Tarabykin V. (2020). Molecular evolution, neurodevelopmental roles and clinical significance of HECT-Type UBE3 E3 ubiquitin ligases. *Cells* 9:2455. 10.3390/cells9112455 33182779 PMC7697756

[B8] Arakhamia T. Lee C. E. Carlomagno Y. Duong D. M. Kundinger S. R. Wang K. (2020). Posttranslational modifications mediate the structural diversity of Tauopathy strains. *Cell* 180 633–644.e12. 32032505 10.1016/j.cell.2020.01.027 PMC7491959

[B9] Balendra R. Isaacs A. M. (2018). C9orf72-mediated ALS and FTD: multiple pathways to disease. *Nat. Rev. Neurol.* 14 544–558. 10.1038/s41582-018-0047-2 30120348 PMC6417666

[B10] Bard J. A. M. Goodall E. A. Greene E. R. Jonsson E. Dong K. C. Martin A. (2018). Structure and function of the 26S Proteasome. *Annu. Rev. Biochem.* 87 697–724. 29652515 10.1146/annurev-biochem-062917-011931 PMC6422034

[B11] Bence N. F. Sampat R. M. Kopito R. R. (2001). Impairment of the ubiquitin-proteasome system by protein aggregation. *Science* 292 1552–1555. 10.1126/science.292.5521.1552 11375494

[B12] Bennett E. J. Bence N. F. Jayakumar R. Kopito R. R. (2005). Global impairment of the ubiquitin-proteasome system by nuclear or cytoplasmic protein aggregates precedes inclusion body formation. *Mol. Cell* 17 351–365. 10.1016/j.molcel.2004.12.021 15694337

[B13] Besche H. C. Haas W. Gygi S. P. Goldberg A. L. (2009). Isolation of mammalian 26S proteasomes and p97/VCP complexes using the ubiquitin-like domain from HHR23B reveals novel proteasome-associated proteins. *Biochemistry* 48 2538–2549. 10.1021/bi802198q 19182904 PMC3811022

[B14] Besche H. C. Sha Z. Kukushkin N. V. Peth A. Hock E. M. Kim W. (2014). Autoubiquitination of the 26S proteasome on Rpn13 regulates breakdown of ubiquitin conjugates. *EMBO J.* 33 1159–1176. 10.1002/embj.201386906 24811749 PMC4193922

[B15] Bjorkoy G. Lamark T. Brech A. Outzen H. Perander M. Overvatn A. (2005). p62/SQSTM1 forms protein aggregates degraded by autophagy and has a protective effect on huntingtin-induced cell death. *J. Cell Biol.* 171 603–614. 10.1083/jcb.200507002 16286508 PMC2171557

[B16] Blythe E. E. Olson K. C. Chau V. Deshaies R. J. (2017). Ubiquitin- and ATP-dependent unfoldase activity of P97/VCP*NPLOC4*UFD1L is enhanced by a mutation that causes multisystem proteinopathy. *Proc. Natl. Acad. Sci. U.S.A.* 114 E4380–E4388. 10.1073/pnas.1706205114 28512218 PMC5465906

[B17] Bodnar N. O. Rapoport T. A. (2017). Molecular mechanism of substrate processing by the Cdc48 ATPase Complex. *Cell* 169 722–735.e9. 10.1016/j.cell.2017.04.020 28475898 PMC5751438

[B18] Bonfanti S. Lionetti M. C. Fumagalli M. R. Chirasani V. R. Tiana G. Dokholyan N. V. (2019). Molecular mechanisms of heterogeneous oligomerization of huntingtin proteins. *Sci. Rep.* 9:7615. 10.1038/s41598-019-44151-0 31110208 PMC6527588

[B19] Braun B. C. Glickman M. Kraft R. Dahlmann B. Kloetzel P. M. Finley D. (1999). The base of the proteasome regulatory particle exhibits chaperone-like activity. *Nat. Cell Biol.* 1 221–226. 10.1038/12043 10559920

[B20] Breydo L. Uversky V. N. (2015). Structural, morphological, and functional diversity of amyloid oligomers. *FEBS Lett.* 589 2640–2648. 10.1016/j.febslet.2015.07.013 26188543

[B21] Brooks P. Fuertes G. Murray R. Z. Bose S. Knecht E. Rechsteiner M. C. (2000). Subcellular localization of proteasomes and their regulatory complexes in mammalian cells. *Biochem. J.* 346(Pt 1) 155–161. 10657252 PMC1220835

[B22] Buchan J. R. Kolaitis R. M. Taylor J. P. Parker R. (2013). Eukaryotic stress granules are cleared by autophagy and Cdc48/VCP function. *Cell* 153 1461–1474. 10.1016/j.cell.2013.05.037 23791177 PMC3760148

[B23] Budenholzer L. Cheng C. L. Li Y. Hochstrasser M. (2017). Proteasome structure and assembly. *J. Mol. Biol.* 429 3500–3524. 28583440 10.1016/j.jmb.2017.05.027 PMC5675778

[B24] Burette A. C. Judson M. C. Li A. N. Chang E. F. Seeley W. W. Philpot B. D. (2018). Subcellular organization of UBE3A in human cerebral cortex. *Mol. Autism* 9:54. 10.1186/s13229-018-0238-0 30364390 PMC6194692

[B25] Bustamante H. A. Gonzalez A. E. Cerda-Troncoso C. Shaughnessy R. Otth C. Soza A. (2018). Interplay between the autophagy-lysosomal pathway and the ubiquitin-proteasome system: a target for therapeutic development in Alzheimer’s disease. *Front. Cell. Neurosci.* 12:126. 10.3389/fncel.2018.00126 29867359 PMC5954036

[B26] Chang L. Monteiro M. J. (2015). Defective proteasome delivery of polyubiquitinated proteins by ubiquilin-2 proteins containing ALS mutations. *PLoS One* 10:e0130162. 10.1371/journal.pone.0130162 26075709 PMC4468220

[B27] Chen R. H. Chen Y. H. Huang T. Y. (2019). Ubiquitin-mediated regulation of autophagy. *J. Biomed. Sci.* 26:80. 10.1186/s12929-019-0569-y 31630678 PMC6802350

[B28] Cherkasov V. Hofmann S. Druffel-Augustin S. Mogk A. Tyedmers J. Stoecklin G. (2013). Coordination of translational control and protein homeostasis during severe heat stress. *Curr. Biol.* 23 2452–2462. 10.1016/j.cub.2013.09.058 24291094

[B29] Choi W. H. de Poot S. A. Lee J. H. Kim J. H. Han D. H. Kim Y. K. (2016). Open-gate mutants of the mammalian proteasome show enhanced ubiquitin-conjugate degradation. *Nat. Commun.* 7:10963. 10.1038/ncomms10963 26957043 PMC4786872

[B30] Chu B. W. Kovary K. M. Guillaume J. Chen L. C. Teruel M. N. Wandless T. J. (2013). The E3 ubiquitin ligase UBE3C enhances proteasome processivity by ubiquitinating partially proteolyzed substrates. *J. Biol. Chem.* 288 34575–34587. 10.1074/jbc.M113.499350 24158444 PMC3843071

[B31] Chuang K. H. Liang F. Higgins R. Wang Y. (2016). Ubiquilin/Dsk2 promotes inclusion body formation and vacuole (lysosome)-mediated disposal of mutated huntingtin. *Mol. Biol. Cell* 27 2025–2036. 10.1091/mbc.E16-01-0026 27170182 PMC4927277

[B32] Cliffe R. Sang J. C. Kundel F. Finley D. Klenerman D. Ye Y. (2019). Filamentous aggregates are fragmented by the proteasome holoenzyme. *Cell Rep.* 26 2140–2149.e3. 10.1016/j.celrep.2019.01.096 30784595 PMC6381791

[B33] Coleman R. A. Mohallem R. Aryal U. K. Trader D. J. (2021). Protein degradation profile reveals dynamic nature of 20S proteasome small molecule stimulation. *RSC Chem. Biol.* 2 636–644. 10.1039/d0cb00191k 34458805 PMC8341874

[B34] Collins G. A. Goldberg A. L. (2017). The logic of the 26S proteasome. *Cell* 169 792–806. 10.1016/j.cell.2017.04.023 28525752 PMC5609836

[B35] Cupo R. R. Shorter J. (2020). Skd3 (human ClpB) is a potent mitochondrial protein disaggregase that is inactivated by 3-methylglutaconic aciduria-linked mutations. *Elife* 9:e55279. 10.7554/eLife.55279 32573439 PMC7343390

[B36] Darwich N. F. Phan J. M. Kim B. Suh E. Papatriantafyllou J. D. Changolkar L. (2020). Autosomal dominant VCP hypomorph mutation impairs disaggregation of PHF-tau. *Science* 370:eaay8826. 10.1126/science.aay8826 33004675 PMC7818661

[B37] De S. Wirthensohn D. C. Flagmeier P. Hughes C. Aprile F. A. Ruggeri F. S. (2019b). Different soluble aggregates of Abeta42 can give rise to cellular toxicity through different mechanisms. *Nat. Commun.* 10:1541. 10.1038/s41467-019-09477-3 30948723 PMC6449370

[B38] De S. Whiten D. R. Ruggeri F. S. Hughes C. Rodrigues M. Sideris D. I. (2019a). Soluble aggregates present in cerebrospinal fluid change in size and mechanism of toxicity during Alzheimer’s disease progression. *Acta Neuropathol. Commun.* 7:120. 10.1186/s40478-019-0777-4 31349874 PMC6659275

[B39] Dedmon M. M. Christodoulou J. Wilson M. R. Dobson C. M. (2005). Heat shock protein 70 inhibits alpha-synuclein fibril formation via preferential binding to prefibrillar species. *J. Biol. Chem.* 280 14733–14740. 10.1074/jbc.M413024200 15671022

[B40] Demishtein A. Fraiberg M. Berko D. Tirosh B. Elazar Z. Navon A. (2017). SQSTM1/p62-mediated autophagy compensates for loss of proteasome polyubiquitin recruiting capacity. *Autophagy* 13 1697–1708. 10.1080/15548627.2017.1356549 28792301 PMC5640208

[B41] Deng H. X. Chen W. Hong S. T. Boycott K. M. Gorrie G. H. Siddique N. (2011). Mutations in UBQLN2 cause dominant X-linked juvenile and adult-onset ALS and ALS/dementia. *Nature* 477 211–215. 10.1038/nature10353 21857683 PMC3169705

[B42] Desantis M. E. Shorter J. (2012). The elusive middle domain of Hsp104 and ClpB: location and function. *Biochim. Biophys. Acta* 1823 29–39. 10.1016/j.bbamcr.2011.07.014 21843558 PMC3219823

[B43] Deville C. Franke K. Mogk A. Bukau B. Saibil H. R. (2019). Two-step activation mechanism of the ClpB disaggregase for sequential substrate threading by the main ATPase motor. *Cell Rep.* 27 3433–3446.e4. 10.1016/j.celrep.2019.05.075 31216466 PMC6593972

[B44] Diaz-Hernandez M. Valera A. G. Moran M. A. Gomez-Ramos P. Alvarez-Castelao B. Castano J. G. (2006). Inhibition of 26S proteasome activity by huntingtin filaments but not inclusion bodies isolated from mouse and human brain. *J. Neurochem.* 98 1585–1596. 10.1111/j.1471-4159.2006.03968.x 16787406

[B45] Dickey C. A. Kamal A. Lundgren K. Klosak N. Bailey R. M. Dunmore J. (2007). The high-affinity HSP90-CHIP complex recognizes and selectively degrades phosphorylated tau client proteins. *J. Clin. Invest.* 117 648–658. 10.1172/JCI29715 17304350 PMC1794119

[B46] Dikic I. (2017). Proteasomal and autophagy degradation systems. *Annu. Rev. Biochem.* 86 1–32. 28460188 10.1146/annurev-biochem-061516-044908

[B47] Eisele F. Eisele-Burger A. M. Hao X. Berglund L. L. Hoog J. L. Liu B. (2021). An Hsp90 co-chaperone links protein folding and degradation and is part of a conserved protein quality control. *Cell Rep.* 35:109328. 10.1016/j.celrep.2021.109328 34192536

[B48] Evans C. G. Wisen S. Gestwicki J. E. (2006). Heat shock proteins 70 and 90 inhibit early stages of amyloid beta-(1-42) aggregation in vitro. *J. Biol. Chem.* 281 33182–33191. 10.1074/jbc.M606192200 16973602

[B49] Fang N. N. Ng A. H. Measday V. Mayor T. (2011). Hul5 HECT ubiquitin ligase plays a major role in the ubiquitylation and turnover of cytosolic misfolded proteins. *Nat. Cell Biol.* 13 1344–1352. 10.1038/ncb2343 21983566 PMC4961474

[B50] Feleciano D. R. Juenemann K. Iburg M. Bras I. C. Holmberg C. I. Kirstein J. (2019). Crosstalk between chaperone-mediated protein disaggregation and proteolytic pathways in aging and disease. *Front. Aging Neurosci.* 11:9. 10.3389/fnagi.2019.00009 30760997 PMC6361847

[B51] Finley D. (2009). Recognition and processing of ubiquitin-protein conjugates by the proteasome. *Annu. Rev. Biochem.* 78 477–513. 10.1146/annurev.biochem.78.081507.101607 19489727 PMC3431160

[B52] Frottin F. Schueder F. Tiwary S. Gupta R. Körner R. Schlichthaerle T. (2019). The nucleolus functions as a phase-separated protein quality control compartment. *Science* 365 342–347. 10.1126/science.aaw9157 31296649

[B53] Fu A. Cohen-Kaplan V. Avni N. Livneh I. Ciechanover A. (2021). p62-containing, proteolytically active nuclear condensates, increase the efficiency of the ubiquitin–proteasome system. *Proc. Natl. Acad. Sci. U.S.A.* 118:e2107321118. 10.1073/pnas.2107321118 34385323 PMC8379982

[B54] Fujiwara H. Hasegawa M. Dohmae N. Kawashima A. Masliah E. Goldberg M. S. (2002). alpha-Synuclein is phosphorylated in synucleinopathy lesions. *Nat. Cell Biol.* 4 160–164. 11813001 10.1038/ncb748

[B55] Furukawa Y. Vigouroux S. Wong H. Guttman M. Rajput A. H. Ang L. (2002). Brain proteasomal function in sporadic Parkinson’s disease and related disorders. *Ann. Neurol.* 51 779–782. 10.1002/ana.10207 12112087

[B56] Ganassi M. Mateju D. Bigi I. Mediani L. Poser I. Lee H. O. (2016). A surveillance function of the HSPB8-BAG3-HSP70 chaperone complex ensures stress granule integrity and dynamism. *Mol. Cell* 63 796–810. 10.1016/j.molcel.2016.07.021 27570075

[B57] Gao X. Carroni M. Nussbaum-Krammer C. Mogk A. Nillegoda N. B. Szlachcic A. (2015). Human Hsp70 disaggregase reverses Parkinson’s-linked alpha-synuclein amyloid fibrils. *Mol. Cell* 59 781–793. 10.1016/j.molcel.2015.07.012 26300264 PMC5072489

[B58] Garcia-Sierra F. Jarero-Basulto J. J. Kristofikova Z. Majer E. Binder L. I. Ripova D. (2012). Ubiquitin is associated with early truncation of tau protein at aspartic acid(421) during the maturation of neurofibrillary tangles in Alzheimer’s disease. *Brain Pathol.* 22 240–250. 10.1111/j.1750-3639.2011.00525.x 21919991 PMC8029281

[B59] Goedert M. Spillantini M. G. (2017). Propagation of Tau aggregates. *Mol. Brain* 10:18. 10.1186/s13041-017-0298-7 PMC5450399 28558799

[B60] Gottlieb C. D. Thompson A. C. S. Ordureau A. Harper J. W. Kopito R. R. (2019). Acute unfolding of a single protein immediately stimulates recruitment of ubiquitin protein ligase E3C (UBE3C) to 26S proteasomes. *J. Biol. Chem.* 294 16511–16524. 10.1074/jbc.RA119.009654 31375563 PMC6851344

[B61] Gu J. Wang C. Hu R. Li Y. Zhang S. Sun Y. (2021). Hsp70 chaperones TDP-43 in dynamic, liquid-like phase and prevents it from amyloid aggregation. *Cell Res.* 31 1024–1027. 10.1038/s41422-021-00526-5 34239072 PMC8410890

[B62] Gu Z. C. Wu E. Sailer C. Jando J. Styles E. Eisenkolb I. (2017). Ubiquitin orchestrates proteasome dynamics between proliferation and quiescence in yeast. *Mol. Biol. Cell* 28 2479–2491. 10.1091/mbc.E17-03-0162 28768827 PMC5597321

[B63] Guerrero C. Milenkovic T. Przulj N. Kaiser P. Huang L. (2008). Characterization of the proteasome interaction network using a QTAX-based tag-team strategy and protein interaction network analysis. *Proc. Natl. Acad. Sci. U.S.A.* 105 13333–13338. 10.1073/pnas.0801870105 18757749 PMC2533190

[B64] Guo Q. Lehmer C. Martínez-Sánchez A. Rudack T. Beck F. Hartmann H. (2018). In Situ structure of neuronal C9orf72 Poly-GA aggregates reveals proteasome recruitment. *Cell* 172 696–705.e12. 10.1016/j.cell.2017.12.030 29398115 PMC6035389

[B65] Hanzelmann P. Schindelin H. (2016). Structural Basis of ATP Hydrolysis and Intersubunit Signaling in the AAA+ ATPase p97. *Structure* 24 127–139. 10.1016/j.str.2015.10.026 26712278

[B66] Hanzelmann P. Schindelin H. (2017). The interplay of cofactor interactions and post-translational modifications in the regulation of the AAA+ ATPase p97. *Front. Mol. Biosci.* 4:21. 10.3389/fmolb.2017.00021 28451587 PMC5389986

[B67] Hao R. Nanduri P. Rao Y. Panichelli R. S. Ito A. Yoshida M. (2013). Proteasomes activate aggresome disassembly and clearance by producing unanchored ubiquitin chains. *Mol. Cell* 51 819–828. 10.1016/j.molcel.2013.08.016 24035499 PMC3791850

[B68] Hernandez-Vega A. Braun M. Scharrel L. Jahnel M. Wegmann S. Hyman B. T. (2017). Local nucleation of microtubule bundles through tubulin concentration into a condensed Tau phase. *Cell Rep.* 20 2304–2312. 10.1016/j.celrep.2017.08.042 28877466 PMC5828996

[B69] Higgins R. Kabbaj M. H. Sherwin D. Howell L. A. Hatcher A. Tomko R. J. Jr. (2020). The Cdc48 complex alleviates the cytotoxicity of misfolded proteins by regulating Ubiquitin homeostasis. *Cell Rep.* 32:107898. 10.1016/j.celrep.2020.107898 32668237 PMC7392062

[B70] Hirabayashi M. Inoue K. Tanaka K. Nakadate K. Ohsawa Y. Kamei Y. (2001). VCP/p97 in abnormal protein aggregates, cytoplasmic vacuoles, and cell death, phenotypes relevant to neurodegeneration. *Cell Death Differ.* 8 977–984. 10.1038/sj.cdd.4400907 11598795

[B71] Hjerpe R. Bett J. S. Keuss M. J. Solovyova A. McWilliams T. G. Johnson C. (2016). UBQLN2 mediates autophagy-independent protein aggregate clearance by the proteasome. *Cell* 166 935–949. 10.1016/j.cell.2016.07.001 27477512 PMC5003816

[B72] Hsu M.-T. Guo C.-L. Liou A. Y. Chang T.-Y. Ng M.-C. Florea B. I. (2015). Stage-dependent axon transport of proteasomes contributes to axon development. *Dev. Cell* 35 418–431. 10.1016/j.devcel.2015.10.018 26609957

[B73] Huang X. Luan B. Wu J. Shi Y. (2016). An atomic structure of the human 26S proteasome. *Nat. Struct. Mol. Biol.* 23 778–785. 10.1038/nsmb.3273 27428775

[B74] Hyman A. A. Weber C. A. Jülicher F. (2014). Liquid-Liquid Phase Separation in Biology. *Annu. Rev. Cell Dev. Biol.* 30 39–58. 25288112 10.1146/annurev-cellbio-100913-013325

[B75] Iljina M. Tosatto L. Choi M. L. Sang J. C. Ye Y. Hughes C. D. (2016). Arachidonic acid mediates the formation of abundant alpha-helical multimers of alpha-synuclein. *Sci. Rep.* 6:33928. 10.1038/srep33928 27671749 PMC5037366

[B76] Jana N. R. Dikshit P. Goswami A. Kotliarova S. Murata S. Tanaka K. (2005). Co-chaperone CHIP associates with expanded polyglutamine protein and promotes their degradation by proteasomes. *J. Biol. Chem.* 280 11635–11640. 10.1074/jbc.M412042200 15664989

[B77] Johnson J. O. Mandrioli J. Benatar M. Abramzon Y. Van Deerlin V. M. Trojanowski J. Q. (2010). Traynor, Exome sequencing reveals VCP mutations as a cause of familial ALS. *Neuron* 68 857–864. 10.1016/j.neuron.2010.11.036 21145000 PMC3032425

[B78] Kalia L. V. Kalia S. K. Chau H. Lozano A. M. Hyman B. T. McLean P. J. (2011). Ubiquitinylation of alpha-synuclein by carboxyl terminus Hsp70-interacting protein (CHIP) is regulated by Bcl-2-associated athanogene 5 (BAG5). *PLoS One* 6:e14695. 10.1371/journal.pone.0014695 21358815 PMC3040167

[B79] Karampetsou M. Ardah M. T. Semitekolou M. Polissidis A. Samiotaki M. Kalomoiri M. (2017). Phosphorylated exogenous alpha-synuclein fibrils exacerbate pathology and induce neuronal dysfunction in mice. *Sci. Rep.* 7:16533. 10.1038/s41598-017-15813-8 29184069 PMC5705684

[B80] Katsuragi Y. Ichimura Y. Komatsu M. (2015). p62/SQSTM1 functions as a signaling hub and an autophagy adaptor. *FEBS J.* 282 4672–4678. 10.1111/febs.13540 26432171

[B81] Keller J. N. Hanni K. B. Markesbery W. R. (2000). Impaired proteasome function in Alzheimer’s disease. *J. Neurochem.* 75 436–439. 10.1046/j.1471-4159.2000.0750436.x 10854289

[B82] Kim H. Kunz P. A. Mooney R. Philpot B. D. Smith S. L. (2016). Maternal loss of Ube3a impairs experience-driven dendritic spine maintenance in the developing visual cortex. *J. Neurosci.* 36 4888–4894. 10.1523/JNEUROSCI.4204-15.2016 27122043 PMC4846678

[B83] Klenerman D. Meng J. Zhang Y. Saman D. De S. Sang J. (2021). Hyperphosphorylated tau self-assembles into amorphous aggregates eliciting TLR4-dependent inflammatory responses. *Res. Sq.* [Preprint]. 10.21203/rs.3.rs-374549/v1 PMC9110413 35577786

[B84] Klosin A. Oltsch F. Harmon T. Honigmann A. Julicher F. Hyman A. A. (2020). Phase separation provides a mechanism to reduce noise in cells. *Science* 367 464–468. 10.1126/science.aav6691 31974256

[B85] Klucken J. Shin Y. Masliah E. Hyman B. T. McLean P. J. (2004). Hsp70 reduces alpha-synuclein aggregation and toxicity. *J. Biol. Chem.* 279 25497–25502. 10.1074/jbc.M400255200 15044495

[B86] Knopman D. S. Amieva H. Petersen R. C. Chetelat G. Holtzman D. M. Hyman B. T. (2021). Alzheimer disease. *Nat. Rev. Dis. Primers* 7:33. 10.1038/s41572-021-00269-y PMC8574196 33986301

[B87] Komander D. Rape M. (2012). The ubiquitin code. *Annu. Rev. Biochem.* 81 203–229. 22524316 10.1146/annurev-biochem-060310-170328

[B88] Kopeikina K. J. Hyman B. T. Spires-Jones T. L. (2012). Soluble forms of tau are toxic in Alzheimer’s disease. *Transl. Neurosci.* 3 223–233. 10.2478/s13380-012-0032-y 23029602 PMC3460520

[B89] Korolchuk V. I. Menzies F. M. Rubinsztein D. C. (2010). Mechanisms of cross-talk between the ubiquitin-proteasome and autophagy-lysosome systems. *FEBS Lett.* 584 1393–1398. 10.1016/j.febslet.2009.12.047 20040365

[B90] Kristiansen M. Deriziotis P. Dimcheff D. E. Jackson G. S. Ovaa H. Naumann H. (2007). Disease-associated prion protein oligomers inhibit the 26S proteasome. *Mol. Cell* 26 175–188. 10.1016/j.molcel.2007.04.001 17466621

[B91] Kuhnle S. Martinez-Noel G. Leclere F. Hayes S. D. Harper J. W. Howley P. M. (2018). Angelman syndrome-associated point mutations in the Zn(2+)-binding N-terminal (AZUL) domain of UBE3A ubiquitin ligase inhibit binding to the proteasome. *J. Biol. Chem.* 293 18387–18399. 10.1074/jbc.RA118.004653 30257870 PMC6254356

[B92] Kumar P. Ambasta R. K. Veereshwarayya V. Rosen K. M. Kosik K. S. Band H. (2007). CHIP and HSPs interact with beta-APP in a proteasome-dependent manner and influence Abeta metabolism. *Hum. Mol. Genet.* 16 848–864. 10.1093/hmg/ddm030 17317785

[B93] Kundel F. De S. Flagmeier P. Horrocks M. H. Kjaergaard M. Shammas S. L. (2018). Hsp70 Inhibits the nucleation and elongation of Tau and sequesters Tau aggregates with high affinity. *ACS Chem. Biol.* 13 636–646. 10.1021/acschembio.7b01039 29300447 PMC6374916

[B94] Lafarga M. Berciano M. T. Pena E. Mayo I. Castaño J. G. Bohmann D. (2002). Clastosome: a subtype of nuclear body enriched in 19S and 20S proteasomes, ubiquitin, and protein substrates of proteasome. *Mol. Biol. Cell* 13 2771–2782. 10.1091/mbc.e02-03-0122 12181345 PMC117941

[B95] Lamark T. Johansen T. (2012). Aggrephagy: selective disposal of protein aggregates by macroautophagy. *Int. J. Cell Biol.* 2012:736905. 10.1155/2012/736905 22518139 PMC3320095

[B96] Li C. Wang X. Li X. Qiu K. Jiao F. Liu Y. (2019). Proteasome inhibition activates autophagy-lysosome pathway associated With TFEB dephosphorylation and nuclear translocation. *Front. Cell. Dev. Biol.* 7:170. 10.3389/fcell.2019.00170 31508418 PMC6713995

[B97] Li Q. Haney M. S. (2020). The role of glia in protein aggregation. *Neurobiol. Dis.* 143:105015. 10.1016/j.nbd.2020.105015 32663608

[B98] Lobanova E. Whiten D. Ruggeri F. S. Taylor C. Kouli A. Xia Z. (2021). Corrigendum to: Imaging protein aggregates in the serum and cerebrospinal fluid in Parkinson’s disease. *Brain* 144:e90. 10.1093/brain/awab346 34410317 PMC9014748

[B99] Lu Y. Wu J. Dong Y. Chen S. Sun S. Ma Y.-B. (2017). Conformational landscape of the p28-bound human proteasome regulatory particle. *Mol. Cell* 67 322–333.e6. 10.1016/j.molcel.2017.06.007 28689658 PMC5580496

[B100] Marshall R. S. Vierstra R. D. (2018). Proteasome storage granules protect proteasomes from autophagic degradation upon carbon starvation. *eLife* 7:19905. 10.7554/eLife.34532 29624167 PMC5947986

[B101] Marshall R. S. Vierstra R. D. (2019). Dynamic regulation of the 26S proteasome: from synthesis to degradation. *Front. Mol. Biosci.* 6:40. 10.3389/fmolb.2019.00040 31231659 PMC6568242

[B102] Mateju D. Franzmann T. M. Patel A. Kopach A. Boczek E. E. Maharana S. (2017). An aberrant phase transition of stress granules triggered by misfolded protein and prevented by chaperone function. *EMBO J.* 36 1669–1687. 10.15252/embj.201695957 28377462 PMC5470046

[B103] McNaught K. S. Belizaire R. Jenner P. Olanow C. W. Isacson O. (2002). Selective loss of 20S proteasome alpha-subunits in the substantia nigra pars compacta in Parkinson’s disease. *Neurosci. Lett.* 326 155–158. 10.1016/s0304-3940(02)00296-3 12095645

[B104] McSwiggen D. T. Mir M. Darzacq X. Tjian R. (2019). Evaluating phase separation in live cells: diagnosis, caveats, and functional consequences. *Genes Dev.* 33 1619–1634. 10.1101/gad.331520.119 31594803 PMC6942051

[B105] Meacham G. C. Patterson C. Zhang W. Younger J. M. Cyr D. M. (2001). The Hsc70 co-chaperone CHIP targets immature CFTR for proteasomal degradation. *Nat. Cell Biol.* 3 100–105. 10.1038/35050509 11146634

[B106] Menzies F. M. Fleming A. Caricasole A. Bento C. F. Andrews S. P. Ashkenazi A. (2017). Autophagy and neurodegeneration: pathogenic mechanisms and therapeutic opportunities. *Neuron* 93 1015–1034. 10.1016/j.neuron.2017.01.022 28279350

[B107] Meyer H. Weihl C. C. (2014). The VCP/p97 system at a glance: connecting cellular function to disease pathogenesis. *J. Cell Sci.* 127 3877–3883. 10.1242/jcs.093831 25146396 PMC4163641

[B108] Miller V. M. Nelson R. F. Gouvion C. M. Williams A. Rodriguez-Lebron E. Harper S. Q. (2005). CHIP suppresses polyglutamine aggregation and toxicity in vitro and in vivo. *J. Neurosci.* 25 9152–9161. 10.1523/JNEUROSCI.3001-05.2005 16207874 PMC6725774

[B109] Mishra A. Godavarthi S. K. Maheshwari M. Goswami A. Jana N. R. (2009). The ubiquitin ligase E6-AP is induced and recruited to aggresomes in response to proteasome inhibition and may be involved in the ubiquitination of Hsp70-bound misfolded proteins. *J. Biol. Chem.* 284 10537–10545. 10.1074/jbc.M806804200 19233847 PMC2667740

[B110] Muchowski P. J. Schaffar G. Sittler A. Wanker E. E. Hayer-Hartl M. K. Hartl F. U. (2000). Hsp70 and hsp40 chaperones can inhibit self-assembly of polyglutamine proteins into amyloid-like fibrils. *Proc. Natl. Acad. Sci. U.S.A.* 97 7841–7846. 10.1073/pnas.140202897 10859365 PMC16632

[B111] Myeku N. Clelland C. L. Emrani S. Kukushkin N. V. Yu W. H. Goldberg A. L. (2016). Tau-driven 26S proteasome impairment and cognitive dysfunction can be prevented early in disease by activating cAMP-PKA signaling. *Nat. Med.* 22 46–53. 10.1038/nm.4011 26692334 PMC4787271

[B112] Nachman E. Wentink A. S. Madiona K. Bousset L. Katsinelos T. Allinson K. (2020). Disassembly of Tau fibrils by the human Hsp70 disaggregation machinery generates small seeding-competent species. *J. Biol. Chem.* 295 9676–9690. 10.1074/jbc.RA120.013478 32467226 PMC7363153

[B113] Nihongaki Y. Furuhata Y. Otabe T. Hasegawa S. Yoshimoto K. Sato M. (2017). CRISPR-Cas9-based photoactivatable transcription systems to induce neuronal differentiation. *Nat. Methods* 14 963–966. 10.1038/nmeth.4430 28892089

[B114] Nillegoda N. B. Kirstein J. Szlachcic A. Berynskyy M. Stank A. Stengel F. (2015). Crucial HSP70 co-chaperone complex unlocks metazoan protein disaggregation. *Nature* 524 247–251. 10.1038/nature14884 26245380 PMC4830470

[B115] Nonaka T. Masuda-Suzukake M. Hosokawa M. Shimozawa A. Hirai S. Okado H. (2018). C9ORF72 dipeptide repeat poly-GA inclusions promote intracellular aggregation of phosphorylated TDP-43. *Hum. Mol. Genet.* 27 2658–2670. 10.1093/hmg/ddy174 29750243

[B116] Oh E. Akopian D. Rape M. (2018). Principles of ubiquitin-dependent signaling. *Annu. Rev. Cell Dev. Biol.* 34 137–162. 10.1146/annurev-cellbio-100617-062802 30110556

[B117] Olzmann J. A. Carvalho P. (2019). Dynamics and functions of lipid droplets. *Nat. Rev. Mol. Cell Biol.* 20 137–155. 30523332 10.1038/s41580-018-0085-z PMC6746329

[B118] Pack C. G. Yukii H. Toh-e A. Kudo T. Tsuchiya H. Kaiho A. (2014). Quantitative live-cell imaging reveals spatio-temporal dynamics and cytoplasmic assembly of the 26S proteasome. *Nat. Commun.* 5:3396. 10.1038/ncomms4396 24598877

[B119] Pakravan D. Orlando G. Bercier V. Van Den Bosch L. (2021). Role and therapeutic potential of liquid-liquid phase separation in amyotrophic lateral sclerosis. *J. Mol. Cell Biol.* 13 15–28. 10.1093/jmcb/mjaa049 32976566 PMC8036000

[B120] Pankiv S. Clausen T. H. Lamark T. Brech A. Bruun J. A. Outzen H. (2007). p62/SQSTM1 binds directly to Atg8/LC3 to facilitate degradation of ubiquitinated protein aggregates by autophagy. *J. Biol. Chem.* 282 24131–24145. 10.1074/jbc.M702824200 17580304

[B121] Park S. Lee J. H. Jeon J. H. Lee M. J. (2018). Degradation or aggregation: the ramifications of post-translational modifications on tau. *BMB Rep.* 51 265–273. 29661268 10.5483/BMBRep.2018.51.6.077 PMC6033068

[B122] Parsell D. A. Kowal A. S. Singer M. A. Lindquist S. (1994). Protein disaggregation mediated by heat-shock protein Hsp104. *Nature* 372 475–478. 10.1038/372475a0 7984243

[B123] Pecho-Vrieseling E. Rieker C. Fuchs S. Bleckmann D. Esposito M. S. Botta P. (2014). Transneuronal propagation of mutant huntingtin contributes to non-cell autonomous pathology in neurons. *Nat. Neurosci.* 17 1064–1072. 10.1038/nn.3761 25017010

[B124] Peran I. Mittag T. (2020). Molecular structure in biomolecular condensates. *Curr. Opin. Struct. Biol.* 60, 17–26. 10.1016/j.sbi.2019.09.007 31790873 PMC7117980

[B125] Peskett T. R. Rau F. O’Driscoll J. Patani R. Lowe A. R. Saibil H. R. (2018). A liquid to solid phase transition underlying pathological huntingtin Exon1 aggregation. *Mol. Cell* 70 588–601.e6. 10.1016/j.molcel.2018.04.007 29754822 PMC5971205

[B126] Peters J. M. Walsh M. J. Franke W. W. (1990). An abundant and ubiquitous homo-oligomeric ring-shaped ATPase particle related to the putative vesicle fusion proteins Sec18p and NSF. *EMBO J.* 9 1757–1767. 10.1002/j.1460-2075.1990.tb08300.x 2140770 PMC551880

[B127] Petrucelli L. Dickson D. Kehoe K. Taylor J. Snyder H. Grover A. (2004). CHIP and Hsp70 regulate tau ubiquitination, degradation and aggregation. *Hum. Mol. Genet.* 13 703–714. 10.1093/hmg/ddh083 14962978

[B128] Poewe W. Seppi K. Tanner C. M. Halliday G. M. Brundin P. Volkmann J. (2017). Parkinson disease. *Nat. Rev. Dis. Primers* 3:17013. 10.1038/nrdp.2017.13 28332488

[B129] Puchades C. Sandate C. R. Lander G. C. (2020). The molecular principles governing the activity and functional diversity of AAA+ proteins. *Nat. Rev. Mol. Cell Biol.* 21 43–58. 10.1038/s41580-019-0183-6 31754261 PMC9402527

[B130] Qian S. B. McDonough H. Boellmann F. Cyr D. M. Patterson C. (2006). CHIP-mediated stress recovery by sequential ubiquitination of substrates and Hsp70. *Nature* 440 551–555. 10.1038/nature04600 16554822 PMC4112096

[B131] Rauch J. N. Chen J. J. Sorum A. W. Miller G. M. Sharf T. See S. K. (2018). Tau Internalization is regulated by 6-O Sulfation on Heparan Sulfate Proteoglycans (HSPGs). *Sci. Rep.* 8:6382. 10.1038/s41598-018-24904-z 29686391 PMC5913225

[B132] Rauch J. N. Luna G. Guzman E. Audouard M. Challis C. Sibih Y. E. (2020). LRP1 is a master regulator of tau uptake and spread. *Nature* 580 381–385. 10.1038/s41586-020-2156-5 32296178 PMC7687380

[B133] Ray S. Singh N. Kumar R. Patel K. Pandey S. Datta D. (2020). α-Synuclein aggregation nucleates through liquid–liquid phase separation. *Nat. Chem.* 12 705–716. 10.1038/s41557-020-0465-9 32514159

[B134] Rennella E. Huang R. Yu Z. Kay L. E. (2020). Exploring long-range cooperativity in the 20S proteasome core particle from Thermoplasma acidophilum using methyl-TROSY-based NMR. *Proc. Natl. Acad. Sci. U.S.A.* 117 5298–5309. 10.1073/pnas.1920770117 32094174 PMC7071895

[B135] Rosenzweig R. Nillegoda N. B. Mayer M. P. Bukau B. (2019). The Hsp70 chaperone network. *Nat. Rev. Mol. Cell Biol.* 20 665–680. 31253954 10.1038/s41580-019-0133-3

[B136] Runwal G. Stamatakou E. Siddiqi F. H. Puri C. Zhu Y. Rubinsztein D. C. (2019). LC3-positive structures are prominent in autophagy-deficient cells. *Sci. Rep.* 9:10147. 10.1038/s41598-019-46657-z 31300716 PMC6625982

[B137] Saffert P. Enenkel C. Wendler P. (2017). Structure and function of p97 and Pex1/6 Type II AAA+ complexes. *Front. Mol. Biosci.* 4:33. 10.3389/fmolb.2017.00033 28611990 PMC5447069

[B138] Schuster B. S. Reed E. H. Parthasarathy R. Jahnke C. N. Caldwell R. M. Bermudez J. G. (2018). Controllable protein phase separation and modular recruitment to form responsive membraneless organelles. *Nat. Commun.* 9:2985. 10.1038/s41467-018-05403-1 30061688 PMC6065366

[B139] Schweighauser M. Shi Y. Tarutani A. Kametani F. Murzin A. G. Ghetti B. (2020). Structures of alpha-synuclein filaments from multiple system atrophy. *Nature* 585 464–469. 32461689 10.1038/s41586-020-2317-6 PMC7116528

[B140] Seo H. Sonntag K. C. Isacson O. (2004). Generalized brain and skin proteasome inhibition in Huntington’s disease. *Ann. Neurol.* 56 319–328. 10.1002/ana.20207 15349858

[B141] Sharkey L. M. Safren N. Pithadia A. S. Gerson J. E. Dulchavsky M. Fischer S. (2018). Mutant UBQLN2 promotes toxicity by modulating intrinsic self-assembly. *Proc. Natl. Acad. Sci. U.S.A.* 115 E10495–E10504. 10.1073/pnas.1810522115 30333186 PMC6217421

[B142] Shin Y. Brangwynne C. P. (2017). Liquid phase condensation in cell physiology and disease. *Science* 357:eaaf4382. 10.1126/science.aaf4382 28935776

[B143] Shin Y. Berry J. Pannucci N. Haataja M. P. Toettcher J. E. Brangwynne C. P. (2017). Spatiotemporal control of intracellular phase transitions using light-activated optoDroplets. *Cell* 168 159–171.e14. 10.1016/j.cell.2016.11.054 28041848 PMC5562165

[B144] Shin Y. Klucken J. Patterson C. Hyman B. T. McLean P. J. (2005). The co-chaperone carboxyl terminus of Hsp70-interacting protein (CHIP) mediates alpha-synuclein degradation decisions between proteasomal and lysosomal pathways. *J. Biol. Chem.* 280 23727–23734. 10.1074/jbc.M503326200 15845543

[B145] Simic G. Babic Leko M. Wray S. Harrington C. Delalle I. Jovanov-Milosevic N. (2016). Tau protein hyperphosphorylation and aggregation in Alzheimer’s disease and other Tauopathies, and possible neuroprotective strategies. *Biomolecules* 6:6. 10.3390/biom6010006 26751493 PMC4808800

[B146] Singh S. Sivaraman J. (2020). Crystal structure of HECT domain of UBE3C E3 ligase and its ubiquitination activity. *Biochem. J.* 477 905–923. 10.1042/BCJ20200027 32039437

[B147] Soto C. Pritzkow S. (2018). Protein misfolding, aggregation, and conformational strains in neurodegenerative diseases. *Nat Neurosci* 21 1332–1340. 10.1038/s41593-018-0235-9 30250260 PMC6432913

[B148] Sun D. Wu R. Zheng J. Li P. Yu L. (2018). Polyubiquitin chain-induced p62 phase separation drives autophagic cargo segregation. *Cell Res.* 28 405–415. 10.1038/s41422-018-0017-7 29507397 PMC5939046

[B149] Sun X. Qiu H. (2020). Valosin-containing protein, a calcium-associated ATPase protein, in endoplasmic reticulum and mitochondrial function and its implications for diseases. *Int. J. Mol. Sci.* 21:3842. 10.3390/ijms21113842 32481679 PMC7312078

[B150] Svilar D. Dyavaiah M. Brown A. R. Tang J. B. Li J. McDonald P. R. (2012). Alkylation sensitivity screens reveal a conserved cross-species functionome. *Mol. Cancer Res.* 10 1580–1596. 10.1158/1541-7786.MCR-12-0168 23038810 PMC3877719

[B151] Swatek K. N. Komander D. (2016). Ubiquitin modifications. *Cell Res.* 26 399–422. 27012465 10.1038/cr.2016.39 PMC4822133

[B152] Tabrizi S. J. Flower M. D. Ross C. A. Wild E. J. (2020). Huntington disease: new insights into molecular pathogenesis and therapeutic opportunities. *Nat. Rev. Neurol.* 16 529–546. 10.1038/s41582-020-0389-4 32796930

[B153] Tai H.-C. Besche H. Goldberg A. L. Schuman E. M. (2010). Characterization of the Brain 26S Proteasome and its Interacting Proteins. *Front. Mol. Neurosci.* 3:12. 10.3389/fnmol.2010.00012 20717473 PMC2901091

[B154] Tao J. Berthet A. Citron Y. R. Tsiolaki P. L. Stanley R. Gestwicki J. E. (2021). Hsp70 chaperone blocks alpha-synuclein oligomer formation via a novel engagement mechanism. *J. Biol. Chem.* 296:100613. 10.1016/j.jbc.2021.100613 33798554 PMC8102405

[B155] Tetzlaff J. E. Putcha P. Outeiro T. F. Ivanov A. Berezovska O. Hyman B. T. (2008). CHIP targets toxic alpha-Synuclein oligomers for degradation. *J. Biol. Chem.* 283 17962–17968. 10.1074/jbc.M802283200 18436529 PMC2936239

[B156] Thibaudeau T. A. Anderson R. T. Smith D. M. (2018). A common mechanism of proteasome impairment by neurodegenerative disease-associated oligomers. *Nat. Commun.* 9:1097. 10.1038/s41467-018-03509-0 29545515 PMC5854577

[B157] Turakhiya A. Meyer S. R. Marincola G. Bohm S. Vanselow J. T. Schlosser A. (2018). ZFAND1 recruits p97 and the 26S proteasome to promote the clearance of arsenite-induced stress granules. *Mol. Cell* 70 906–919.e7. 10.1016/j.molcel.2018.04.021 29804830

[B158] Upadhyay A. Joshi V. Amanullah A. Mishra R. Arora N. Prasad A. (2017). E3 ubiquitin ligases neurobiological mechanisms: development to degeneration. *Front. Mol. Neurosci.* 10:151. 10.3389/fnmol.2017.00151 28579943 PMC5437216

[B159] van Well E. M. Bader V. Patra M. Sanchez-Vicente A. Meschede J. Furthmann N. (2019). A protein quality control pathway regulated by linear ubiquitination. *EMBO J.* 38:e100730. 10.15252/embj.2018100730 30886048 PMC6484417

[B160] van Wijk S. J. Fulda S. Dikic I. Heilemann M. (2019). Visualizing ubiquitination in mammalian cells. *EMBO Rep.* 20:e46520. 10.15252/embr.201846520 30665942 PMC6362358

[B161] Varshavsky A. (2017). The ubiquitin system, autophagy, and regulated protein degradation. *Annu. Rev. Biochem.* 86 123–128. 10.1146/annurev-biochem-061516-044859 28654326

[B162] Vasili E. Dominguez-Meijide A. Outeiro T. F. (2019). Spreading of alpha-synuclein and Tau: a systematic comparison of the mechanisms involved. *Front. Mol. Neurosci.* 12:107. 10.3389/fnmol.2019.00107 31105524 PMC6494944

[B163] Vaz B. Halder S. Ramadan K. (2013). Role of p97/VCP (Cdc48) in genome stability. *Front. Genet.* 4:60. 10.3389/fgene.2013.00060 23641252 PMC3639377

[B164] Wang B. Zhang L. Dai T. Qin Z. Lu H. Zhang L. (2021). Liquid–liquid phase separation in human health and diseases. *Signal Transduct. Target. Ther.* 6:290. 10.1038/s41392-021-00678-1 34334791 PMC8326283

[B165] Watanabe S. Inami H. Oiwa K. Murata Y. Sakai S. Komine O. (2020). Aggresome formation and liquid-liquid phase separation independently induce cytoplasmic aggregation of TAR DNA-binding protein 43. *Cell Death Dis.* 11:909. 10.1038/s41419-020-03116-2 33097688 PMC7585435

[B166] Watts G. D. Wymer J. Kovach M. J. Mehta S. G. Mumm S. Darvish D. (2004). Inclusion body myopathy associated with Paget disease of bone and frontotemporal dementia is caused by mutant valosin-containing protein. *Nat. Genet.* 36 377–381. 10.1038/ng1332 15034582

[B167] Wegmann S. Eftekharzadeh B. Tepper K. Zoltowska K. M. Bennett R. E. Dujardin S. (2018). Tau protein liquid-liquid phase separation can initiate tau aggregation. *EMBO J.* 37:e98049. 10.15252/embj.201798049 29472250 PMC5881631

[B168] Wegmann S. Nicholls S. Takeda S. Fan Z. Hyman B. T. (2016). Formation, release, and internalization of stable tau oligomers in cells. *J. Neurochem.* 139 1163–1174. 10.1111/jnc.13866 27731899 PMC5283951

[B169] Wei M. T. Elbaum-Garfinkle S. Holehouse A. S. Chen C. C. Feric M. Arnold C. B. (2017). Phase behaviour of disordered proteins underlying low density and high permeability of liquid organelles. *Nat. Chem.* 9 1118–1125. 10.1038/nchem.2803 29064502 PMC9719604

[B170] Whiten D. R. Zuo Y. Calo L. Choi M. L. De S. Flagmeier P. (2018). Nanoscopic characterisation of individual endogenous protein aggregates in human neuronal cells. *ChemBioChem* 19 2033–2038. 10.1002/cbic.201800209 30051958 PMC6220870

[B171] Wolf D. H. Stolz A. (2012). The Cdc48 machine in endoplasmic reticulum associated protein degradation. *Biochim. Biophys. Acta* 1823 117–124. 10.1016/j.bbamcr.2011.09.002 21945179

[B172] Wolozin B. Ivanov P. (2019). Stress granules and neurodegeneration. *Nat. Rev. Neurosci.* 20 649–666. 31582840 10.1038/s41583-019-0222-5 PMC6986315

[B173] Yasuda S. Tsuchiya H. Kaiho A. Guo Q. Ikeuchi K. Endo A. (2020). Stress- and ubiquitylation-dependent phase separation of the proteasome. *Nature* 578 296–300. 10.1038/s41586-020-1982-9 32025036

[B174] Ye Y. Klenerman D. Finley D. (2020). N-terminal ubiquitination of amyloidogenic proteins triggers removal of their oligomers by the proteasome holoenzyme. *J. Mol. Biol.* 432 585–596. 10.1016/j.jmb.2019.08.021 31518613 PMC6990400

[B175] Yi J. J. Paranjape S. R. Walker M. P. Choudhury R. Wolter J. M. Fragola G. (2017). The autism-linked UBE3A T485A mutant E3 ubiquitin ligase activates the Wnt/beta-catenin pathway by inhibiting the proteasome. *J. Biol. Chem.* 292 12503–12515. 10.1074/jbc.M117.788448 28559284 PMC5535025

[B176] Yokom A. L. Gates S. N. Jackrel M. E. Mack K. L. Su M. Shorter J. (2016). Spiral architecture of the Hsp104 disaggregase reveals the basis for polypeptide translocation. *Nat. Struct. Mol. Biol.* 23 830–837. 10.1038/nsmb.3277 27478928 PMC5509435

[B177] Zaffagnini G. Savova A. Danieli A. Romanov J. Tremel S. Ebner M. (2018). p62 filaments capture and present ubiquitinated cargos for autophagy. *EMBO J.* 37:e98308. 10.15252/embj.201798308 29343546 PMC5830917

[B178] Zbinden A. Perez-Berlanga M. De Rossi P. Polymenidou M. (2020). Phase separation and neurodegenerative diseases: a disturbance in the force. *Dev. Cell* 55 45–68. 10.1016/j.devcel.2020.09.014 33049211

[B179] Zeiler M. Straube W. L. Lundberg E. Uhlen M. Mann M. (2012). A protein epitope signature tag (PrEST) library allows SILAC-based absolute quantification and multiplexed determination of protein copy numbers in cell lines. *Mol. Cell Proteomics* 11:O111.009613. 10.1074/mcp.O111.009613 21964433 PMC3316735

[B180] Zhang J. Li X. Li J. D. (2019). The roles of post-translational modifications on alpha-Synuclein in the pathogenesis of Parkinson’s Diseases. *Front. Neurosci.* 13:381. 10.3389/fnins.2019.00381 31057362 PMC6482271

[B181] Zhang K. Wang A. Zhong K. Qi S. Wei C. Shu X. (2021). UBQLN2-HSP70 axis reduces poly-Gly-Ala aggregates and alleviates behavioral defects in the C9ORF72 animal model. *Neuron* 109 1949–1962.e6. 10.1016/j.neuron.2021.04.023 33991504

[B182] Zhang S. Hu Z. W. Mao C. Y. Shi C. H. Xu Y. M. (2020). CHIP as a therapeutic target for neurological diseases. *Cell Death Dis.* 11:727. 10.1038/s41419-020-02953-5 32908122 PMC7481199

[B183] Zhang Y. Lippert A. H. Lee J. E. Carr A. R. Ponjavic A. Lee S. F. (2018). Membrane potential regulates the dynamic localisation of mammalian proteasomes. *bioRxiv* [Preprint]. 487702,

[B184] Zheng Q. Huang T. Zhang L. Zhou Y. Luo H. Xu H. (2016). Dysregulation of Ubiquitin-proteasome system in neurodegenerative diseases. *Front. Aging Neurosci.* 8:303. 10.3389/fnagi.2016.00303 28018215 PMC5156861

[B185] Zuo Y. Chong B. K. Jiang K. Finley D. Klenerman D. Ye Y. (2020). A general in vitro assay for studying enzymatic activities of the ubiquitin system. *Biochemistry* 59 851–861. 10.1021/acs.biochem.9b00602 31951392

